# Gastric Cancer Staging with Dual Energy Spectral CT Imaging

**DOI:** 10.1371/journal.pone.0053651

**Published:** 2013-02-12

**Authors:** Zilai Pan, Lifang Pang, Bei Ding, Chao Yan, Huan Zhang, Lianjun Du, Baisong Wang, Qi Song, Kemin Chen, Fuhua Yan

**Affiliations:** 1 Department of Radiology, Ruijin Hospital, Shanghai Jiaotong University School of Medicine, Shanghai, China; 2 Department of Surgeon, Ruijin Hospital, Shanghai Jiaotong University School of Medicine, Shanghai, China; 3 Department of Biomedical Statistics, Shanghai Jiaotong University School of Medicine, Shanghai, China; Virginia Commonwealth University, United States of America

## Abstract

**Purpose:**

To evaluate the clinical utility of dual energy spectral CT (DEsCT) in staging and characterizing gastric cancers.

**Materials and Methods:**

96 patients suspected of gastric cancers underwent dual-phasic scans (arterial phase (AP) and portal venous phase (PP)) with DEsCT mode. Three types of images were reconstructed for analysis: conventional polychromatic images, material-decomposition images, and monochromatic image sets with photon energies from 40 to 140 keV. The polychromatic and monochromatic images were compared in TNM staging. The iodine concentrations in the lesions and lymph nodes were measured on the iodine-based material-decomposition images. These values were further normalized against that in aorta and the normalized iodine concentration (nIC) values were statistically compared. Results were correlated with pathological findings.

**Results:**

The overall accuracies for T, N and M staging were (81.2%, 80.0%, and 98.9%) and (73.9%, 75.0%, and 98.9%) determined with the monochromatic images and the conventional kVp images, respectively. The improvement of the accuracy in N-staging using the keV images was statistically significant (p<0.05). The nIC values between the differentiated and undifferentiated carcinoma and between metastatic and non-metastatic lymph nodes were significantly different both in AP (p = 0.02, respectively) and PP (p = 0.01, respectively). Among metastatic lymph nodes, nIC of the signet-ring cell carcinoma were significantly different from the adenocarcinoma (p = 0.02) and mucinous adenocarcinoma (p = 0.01) in PP.

**Conclusion:**

The monochromatic images obtained with DEsCT may be used to improve the N-staging accuracy. Quantitative iodine concentration measurements may be helpful for differentiating between differentiated and undifferentiated gastric carcinoma, and between metastatic and non-metastatic lymph nodes.

## Introduction

Gastric cancer is one of the most common cancers worldwide with approximately 989,600 new cases and 738,000 deaths per year, accounting for about 8 percent of new cancers [1]. A good prognosis for patients with this disease requires choosing the correct therapy, and making the right therapeutic choice requires accurate preoperative staging [2]–[7]. The recent development of multi-detector row CT (MDCT) scanner has allowed imaging with a thinner section collimation, translating into increased quality on transverse computed tomography scans and multiplanar reconstruction, contributing to the improved accuracy of TNM staging [2]–[3], [8]–[11]. Nowaday MDCT has been widely used in preoperative staging of gastric cancer. However there are still some controversial problems.

Regarding the T-staging, the results from previous reports on the usefulness of CT for T-staging of gastric cancer have shown large variations (overall accuracy rates of 43–82% [12]–[15]. Over-diagnosis sometimes happens when the interface of the lesion and peripheral tissue is blurred by an inflammatory reaction.

Aside from tumor location and depth of infiltration, lymph node status is of particular interest in the pretherapeutic staging of tumors, especially to establish different therapeutic strategies. In early gastric cancer the presence or absence of lymph-node metastases is a critical determinant of whether less invasive treatment, such as endoscopic mucosal resection, can be performed [4]. In advanced carcinoma, lymph node status is an important prognostic factor not only regarding long-term survival, but also planning the optimal extent of lymphadenectomy [16]. In terms of simplicity, reproducibility, homogeneity, and prognostic relevance after gastrectomy, the sixth edition of the International Union Against Cancer (UICC)/American Joint Committee on Cancer (AJCC) staging system, which is the current standard for determining pathologic stage, bases pathologic nodal status on the number of lymph nodes involved [15], [17]–[22]. It differs from criteria used by earlier investigators, who followed Japanese guidelines defined in the General Rules for Gastric Cancer Study in Surgery and Pathology [19]–[20]. Nowadays a precise count of lymph nodes poses a great challenge to the radiologist. Criteria for lymph node malignancy have been controversial [23]–[25]. There has been no worldwide consensus regarding lymph nodes pathology in terms of measuring method (short or long axis), size, shape, or enhancement patterns [26]. The sensitivity and specificity of MDCT for lymph nodes detection varied between 62.5% and 91.9% (median 80.0%) and 50.0% and 87.9% (median 77.8%) [27], which is not satisfactory.

Another impetus of the present study was to find the prognostic indicator preoperatively and untraumatically, and the prognosis is determined by tumor histology, infiltration, extension and stage, especially the TNM system from the AJCC. There is not yet any satisfactory imaging modality to predict prognosis. The advent of MDCT systems enabled perfusion scans to be performed, thus broadening the technique's availability, allowing the measurement of tumor vascular physiology in brain, lung, liver, neck, breast and gastric [28]–[32]. It could be useful for diagnosis, risk-stratification and therapeutic monitoring [33]–[34]. However radiation dose is also a great obstacle and it is also difficult for radiologists to get the perfusion data and accurate TNM staging simultaneously.

Recently, dual energy spectral CT (DEsCT), a new dual energy CT scanning mode based on the rapid switching between high- and low-energy data sets from view to view was introduced. This scanning mode enables precise registration of data sets for the creation of virtual monochromatic spectral images and accurate material- decomposition images (eg, water- and iodine-based material-decomposition images) for quantitative iodine concentration measurement. Material-decomposition(MD) Images are reconstructed from projections created through the material decomposition of the low and high kVp projections. MD Images represent the amount or density of two materials that would be needed to produce the measured attenuation in the 80 kVp and 140 kVp projections. A monochromatic image depicts how the imaged object would look if the x-ray source produced only X-ray photons at a single energy. Therefore, the monochromatic images have reduced beam-hardening effects. The energy level of the monochromatic image sets can be further adjusted to optimally reduce any remaining beam-hardening artifacts, and offer higher contrast-to-noise. The dual energy spectral CT imaging has found its clinical use in cancer differentiation and characterization in the liver [35]–[36]. However, to date, there are few studies dealing with the use of DEsCT for the diagnostic work-up of preoperative TNM staging in gastric cancer. Our study was designed to evaluate the clinical utility of DEsCT imaging in staging and characterizing gastric cancers.

## Materials and Methods

### Patients

The study was approved by our Hospital Ethics Committee. Informed consent was obtained from each patient before imaging. From March 2010 to September 2010, CT was performed in 148 cases of gastric cancer whose diagnoses were confirmed by biopsy. Among them, 46 cases were received neoadjuvant chemotherapy and 6 cases were not operated on due to the severe complications. The 96 gastric cancer cases that were operated on within two weeks (ranging from 4 to 14 days) after CT scans were enrolled in this study. This included 59 men and 37 women. Their mean age was 57 y (range 28–78). Patient records on pathological findings and the pertinent clinical data were shown in Table 1. Gastric carcinomas were classified as differentiated or undifferentiated [16] based on the pathological analysis of the surgical specimen: Papillary and tubular adenocarcinomas were considered differentiated, whereas poorly differentiated adenocarcinoma and signet ring cell carcinoma were considered undifferentiated. Mucinous carcinoma was considered differentiated or undifferentiated depending on other predominant characteristics (papillary, tubular, poorly differentiated, or signet ring cell elements). According to JGCA Japanese classification of gastric cancer 2^nd^ edition (1998) [37], D1 lymphadenectomy involves the dissection of perigastric nodes attached directly to the stomach (compartment I), whereas D2 lymphadenectomy involves complete dissection of compartments I and II. 79 cases underwent gastroectomy with D2, 2 cases of intramucosal carcinoma underwent exploratory laparoscopy with D1 lymphadenectomy, 15 underwent palliative resection. For patients who underwent palliative resection, any obvious enlarged lymph nodes prone to metastasize were resected as much as possible. The surgeon (C Yan) first studied the CT images carefully to map the lymph nodes, and marked them intraoperatively according to the findings on CT images. Locations of lymph nodes were recorded according to the Japanese classification of gastric cancers [38]. To improve the certainty that the lymph nodes seen on CT were accurately correlated with the lymph nodes on surgery, only the lymph nodes larger than 6 mm were included in this study for N-staging.

**Table 1:** Clinical characteristics of the 96 patients.

	Statistics
Age (years)	57
Gender	
Male	59 (61.46%)
Female	37 (38.54%)
BMI	24.82±2.73 kg/m2
Size of tumor	1.5∼8.5cm (mean 3.8cm)
Tumor site	
Fundus	12 (12.5%)
Body	15 (15.6%)
Antrum	41 (42.7%)
Body plus antrum	15 (15.6%)
Fundus plus body	21 (21.9%)
Whole stomach	2 (2.0%)
Histological classification	
Well differentiated adenocarcinoma	1 (0.1%)
Moderately differentiated adenocarcinoma	30 (31.5%)
Poorly differentiated adenocarcinoma	37(38.5%)
Signet-ring cell carcinoma	16 (16.67%)
Mucinous adenocarcinoma	12 (12.5%)

### CT SCAN

After abrosia for 8 h, patients were asked to ingest 1000 ml of water and were injected with a hypotonic agent (20 mg of scopolamine) 10 minutes before the examination. Patients were placed in the supine position. CT was performed with a high-definition CT scanner (Discovery CT750HD, GE Healthcare, Wisconsin, USA). The dual-phasic contrast-enhanced scans were performed using the dual energy spectral CT mode (GSI mode) with a single tube, fast kilo-voltage switching between 80 kVp and 140 kVp in less than 0.5 msec. The other scan parameters included: collimation thickness of 5 mm with 40 mm detector coverage, helical pitch of 0.984, tube current of 600 mA, rotation speed of 0.6 second. The CT dose index volume (CTDIvol) for the dual energy spectral mode for abdominal tumors was 21.84 mGy. Patients were injected with contrast media (iopromide, Ultravisr300; Schering, Berlin, Germany) through power injector (Urich REF XD 2060-Touch, Germany) at a flow rate of 3 ml/s a total of 85∼110 ml (1.5 ml per kilogram of body weight) was injected intravenously. The dual-phasic scans were obtained at 40 s (arterial phase) covering the whole stomach, and 70 s (portal venous phase) covering the whole abdomen and pelvis to detect metastasis and distal lymph node enlargement, after the start of the contrast injection, respectively.

**Figure 1:**
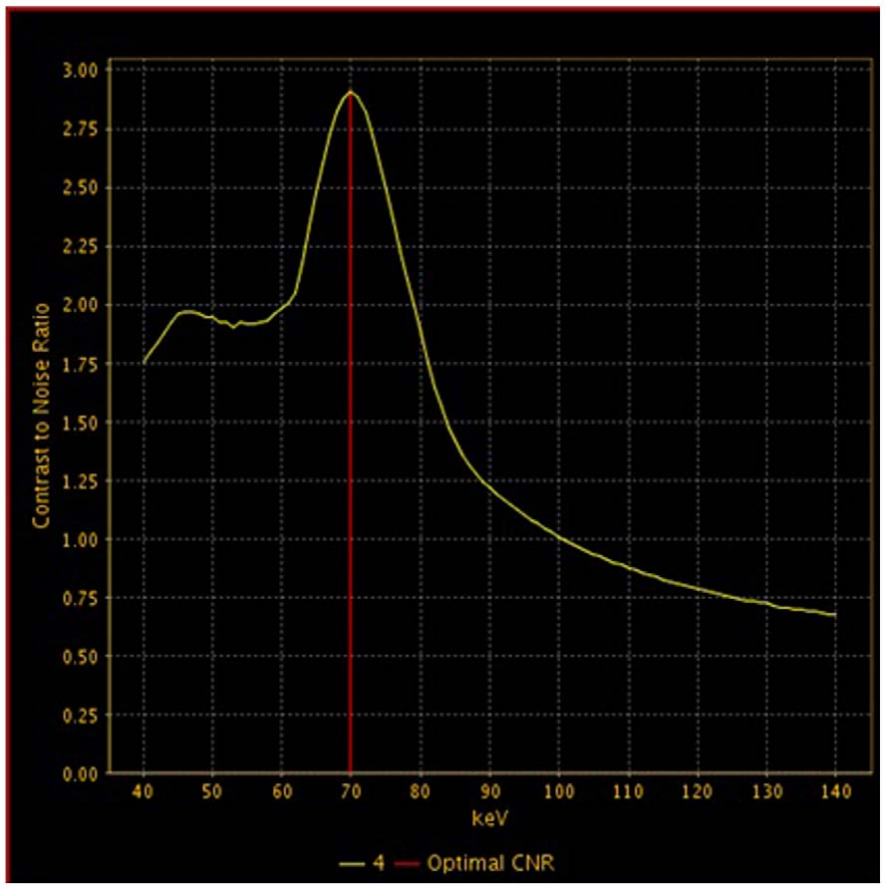
Selecting the best contrast-noise-ratio (CNR) for displaying gastric cancer with GSI Viewer analysis tool. Optimal monochromatic energy of 70 keV achieved the best CNR for the primary lesion.

**Figure 2:**
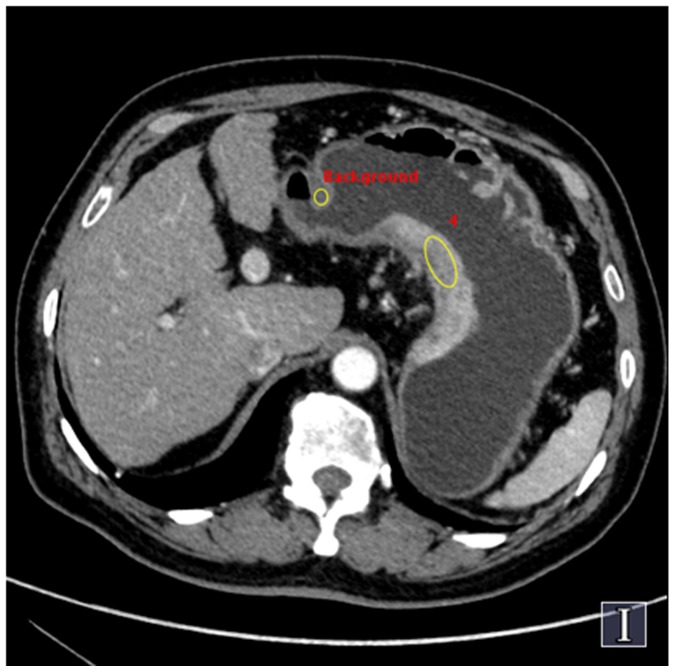
Selecting the best contrast-noise-ratio (CNR) for displaying gastric cancer with GSI Viewer analysis tool. ROI selections for primary lesion and normal gastric wall on an axial image.

Images were reconstructed with a 40 cm display field-of-view (DFOV), 512×512 reconstruction matrix size and a standard reconstruction kernel. Three types of images were reconstructed from the single DEsCT acquisition for analysis: a set of polychromatic images corresponding to the conventional 140 kVp imaging, water- and iodine-based material-decomposition images, and monochromatic image sets corresponding to photon energies ranging from 40 to 140 keV. The spectral CT images were analyzed with the GSI Viewer software 4.4 (GE Healthcare, Waukesha, Wisconsin), with a standard soft-tissue display window preset (WL 40 and WW 400). From the monochromatic image sets, an operation was first made to obtain an optimal energy level (keV) to provide the best contrast-to-noise ratio (CNR) between the gastric lesion and gastric wall. In order to get the optimal keV images, two circular regions-of-interest (ROI) were placed by a radiologist on the lesion and the normal gastric wall. The GSI Viewer software package automatically calculated and displayed the CNR values for the 101 sets of monochromatic images real time. From the CNR plot, the optimal single energy (keV) level for generating the best CNR between the lesion and the normal gastric wall could be selected (Fig. 1–2). Images with 2.5 mm slice thickness and 1.25 mm interval were generated at the optimal keV level and were used for the final TNM staging.

### Image interpretation

In order to make sure for the double-blinded nature of the study, after trained and tested under the uniform standards, four independent radiologists who specialized in gastrointestinal imaging blinded to the endoscopic results were divided into two groups. In each group, two radiologists interpreted the images independent of each other, and disagreement on diagnosis was resolved by consensus. Their results were correlated with pathological findings by two other radiologists.

Group A (Two radiologists with 22 years and 10 years of experience in gastrointestinal imaging, respectively) reviewed all the kVp images (cross section and multiplaner reconstruction, MPR) for TNM staging on AW Volumeshare2 (GE, healthcare), including depth of invasion, presence of lymph node metastasis and distal metastasis. Group B (Two radiologists with 20 years and 10 years of experience in gastrointestinal imaging, respectively) reviewed all the water- and iodine-based material-decomposition images, and the optimal monochromatic images with GSI Viewer software. The optimal monochromatic images were used for the cancer staging. In both groups, MPVR (coronal, oblique or sagital with slice thickness 3 mm) images were made if necessary.

The material decomposition images were used to measure iodine concentrations (IC, in milligrams per milliliter) in the cancers, lymph nodes and aorta. The ROI was drawn on the monochromatic image and copied to the iodine image. The ROI drawn over the tumor, was as large as possible to reduce noise (>50 pixels), with care to exclude peripheral fat and necrotic area. The iodine concentration data in ROI were exported in the excel form. To ensure consistency, an average of two to three separate ROIs over consecutive image slices was obtained. Radiologists in Group B accomplished this process together, disagreement on measurements was resolved by consensus. In order to minimize variations between patients, the iodine concentrations in lesions and lymph nodes were normalized to the iodine concentration in the aorta by dividing the iodine concentration of lesions or lymph nodes to that of aorta to derive a normalized iodine concentration (nIC = IC_lesion_/IC_aorta_).

### Definitions used for staging gastric tumors

Definition for TNM staging by CT was based on the sixth edition of the UICC TNM classification [39] (Table 2). The diagnostic criteria of N staging by D'Elia F were followed: the regional lymph nodes were considered involved when the short-axis diameter was 6 mm for the perigastric lymph nodes and greater than 8 mm for the extraperigastric lymph nodes [12].

**Table 2:** Criteria for TNM staging by CT.

T-staging	**T0** – no detectable primary lesions at the stomach wall;
	**T1** – Enhancing tumor that does not penetrate the submucosal layer;
	**T2** – Transmural enhancement with focal wall thickening in a single-layer pattern, or both abnormal enhancement and abrupt obliteration of the middle layer in a three-layer pattern, or of the outer layer in a two-layer pattern; smooth outer border of the thickened gastric wall or a few small linear strands of soft tissue extending into the fat plane;
	**T3** – Reticular or irregular outer border of the thickened gastric wall or a blurred fat plane around the lesion;
	**T4** – gross infiltration of adjacent organs [12].
N-staging	Numbers of metastatic lymph nodes on images were counted. Regional lymph nodes were considered involved when the short-axis diameter was 6 mm for the perigastric lymph nodes and greater than 8 mm for the extraperigastric lymph nodes [12].
	**N1**, metastases in 1–6 regional lymph nodes;
	**N2**, metastases in 7–15 regional lymph nodes
	**N3**, metastases in >15 regional lymph nodes
M-staging	**M0** – no metastasis; **M1** – distant lymph nodes involved and formation of liver or peritoneal metastasis.

### Statistical analysis

The overall accuracy of CT for T/N/M staging with the conventional kVp images and the optimal monochromatic images were calculated. The difference between the two imaging methods was examined by McNemar test.

The nIC values (including lesion, lymph nodes) at the AP and PP are expressed as mean ± standard deviation (SD). Data was subjected to a Kolmogorov-Smirnov normality test. All findings were prospectively analyzed and correlated with the clinic-pathological results (histological grading, pathological classification and presence of lymph node metastasis). Independent t test and analysis of variance (ANOVA) were calculated and Receiver Operating Characteristic (ROC) curves were derived and used to assist the establishment of the thresholds for the parameter of nIC in differentiating between metastatic and non-metastatic lymph nodes with statistical significance. In the two tailed tests, a p value less than 0.05 was considered statistically significant. All statistics were implemented using SPSS 13.0 software (SPSS Inc, Chicago, IL).

## Results

The gender, age, pathological staging and treatment of the 96 patients were reported in the Appendix S1. Table 1 summarizes the clinical characteristics of the 96 patients. 12 patients had tumor in the fundus, 15 in the body, 41 in the antrum, 15 in both antrum and body, 21 in both fundus and body, and 2 for the whole stomach. On histological classification, 31 patients were diagnosed as having well-differentiated to moderately-well differentiated adenocarcinoma; 37 patients as having poorly differentiated adenocarcinomas, 16 patients as having signet-ring cell carcinoma and 12 patients as having mucinous adenocarcinoma (**Table 1**).

### Comparative study in TNM staging between kVp images and optimal monochromatic keV images

The mean optimal keV for displaying gastric cancer in our patient population was 72±5 keV. Compared to the histopathologic staging, the overall accuracies for T-staging were 81.2% and 73.9% determined with the optimal keV images and the conventional kVp images, respectively. The accuracy of CT for tumor staging with kVp images was 92.7% (89 of 96 patients) for T1, 86.5% (83 of 96 patients) for T2, 80.2% (77 of 96 patients) for T3, and 88.5% (85 of 96 patients) for T4 tumors. These values with the optimal monochromatic keV images were 95.8% (92 of 96 patients) for T1, 89.6% (86 of 96 patients) for T2, 85.4% (82 of 96 patients) for T3, and 91.7% (88 of 96 patients) for T4 tumors (Table 3). McNemar test revealed no significant difference between them (p = 0.153). For patients with non metastatic disease, the accuracy, sensitivity and specificity for this distinction of Tis-T1 tumors vs. T2-3 tumors were 90.4%(66/73), 96.8%(62/64), 44.4%(4/9) with kVp images and 94.5%(69/73), 98.4%(63/64), 66.7%(6/9) with keV images.

**Table 3:** Accuracies, sensitivities and specificities for T staging using kVp images (Group A) and optimal monochromatic images (Group B) with histological examination as the reference standard.

		Histological staging						
CT Staging		T0-1 (n = 9)	T2 (n = 19)	T3 (n = 48)	T4 (n = 20)	Accuracy (%)	Sensitivity (%)	Specificity (%)
A	T0-1	4	2	0	0	92.7	44.4	97.7
	T2	2	14	4	2	86.4	73.7	89.6
	T3	3	3	38	3	80.2	79.2	81.2
	T4	0	0	6	15	88.5	75	92.1
B	T0-1	6	1	0	0	95.8	66.7	98.8
	T2	2	17	5	1	89.6	89.5	89.6
	T3	1	1	39	3	85.4	81.3	89.6
	T4	0	0	4	16	91.7	80	94.7

T1–T4 data are presented as the number of patients; other variables as percentages.

**Figure 3:**
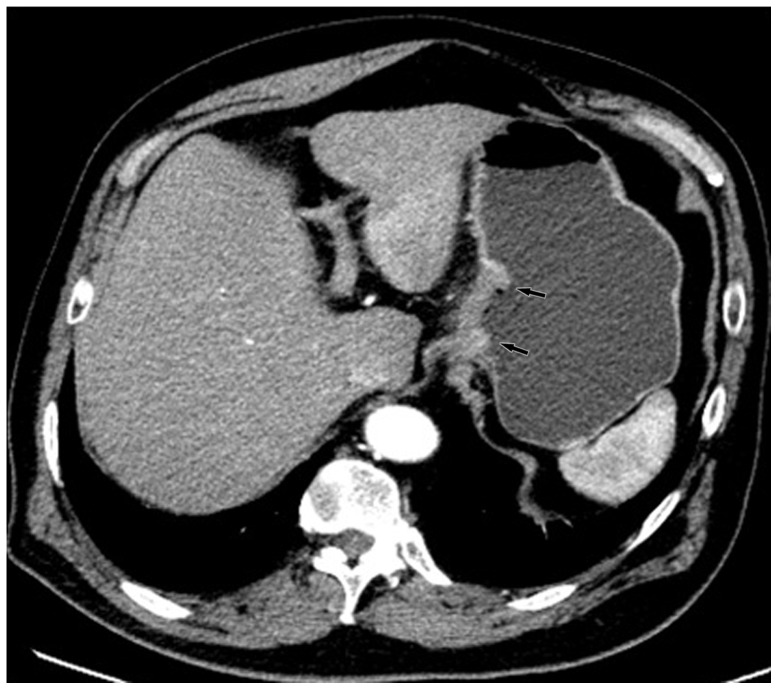
Monochromatic image in arterial phase of a 48-year-old man with stage T3N2 gastric adenocarcinoma demonstrated focal thickening (arrows) of the cardiac portion and lesser curvature with the abruptly interruption of mucous enhancement.

**Figure 4:**
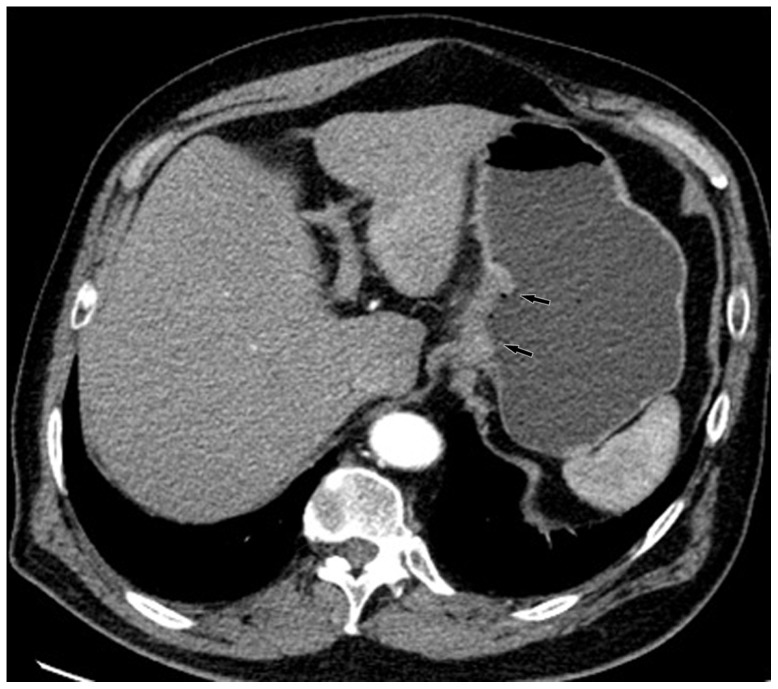
Same patient as Figure 3. KVp image in arterial phase maintained a relatively sharp tumor contour.

**Figure 5:**
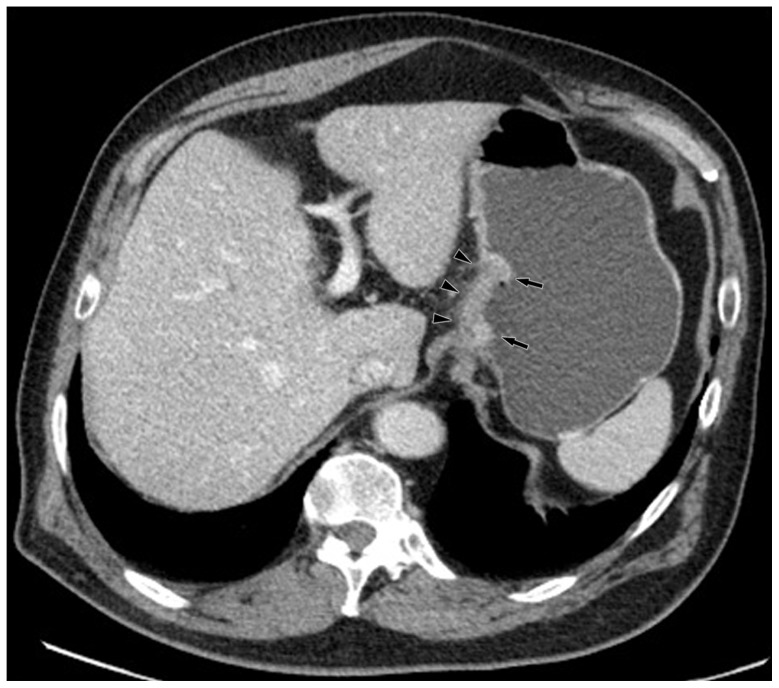
Same patient as Figure 3. Monochromatic image in portal phase demonstrated striation enhancement of blurring and wide reticular strands surrounding the outer border (arrow heads) of the tumor staged as T3 which was proved by histology.

**Figure 6:**
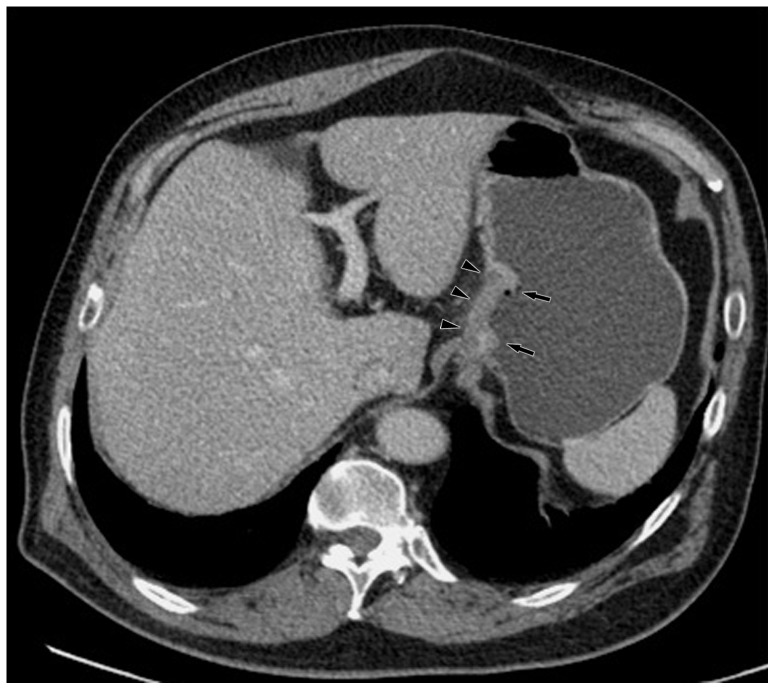
Same patient as Figure 3. KVp image in portal phase maintained a relatively sharp tumor contour and a clear stomach fat plane (arrow heads), staged as T2.

**Figure 7:**
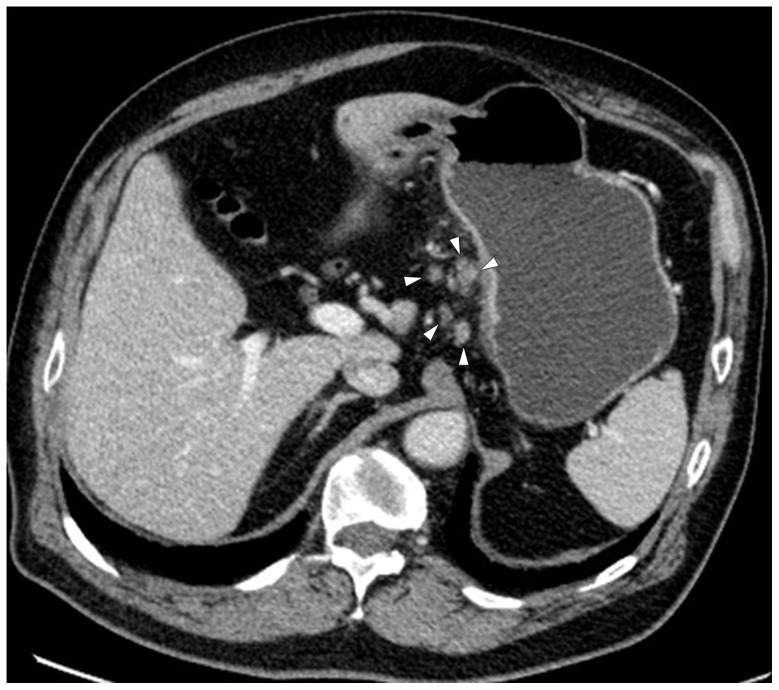
Same patient as Figure 3. Five lymph nodes (arrow heads) were found in lesser curvature with monochromatic images.

**Figure 8:**
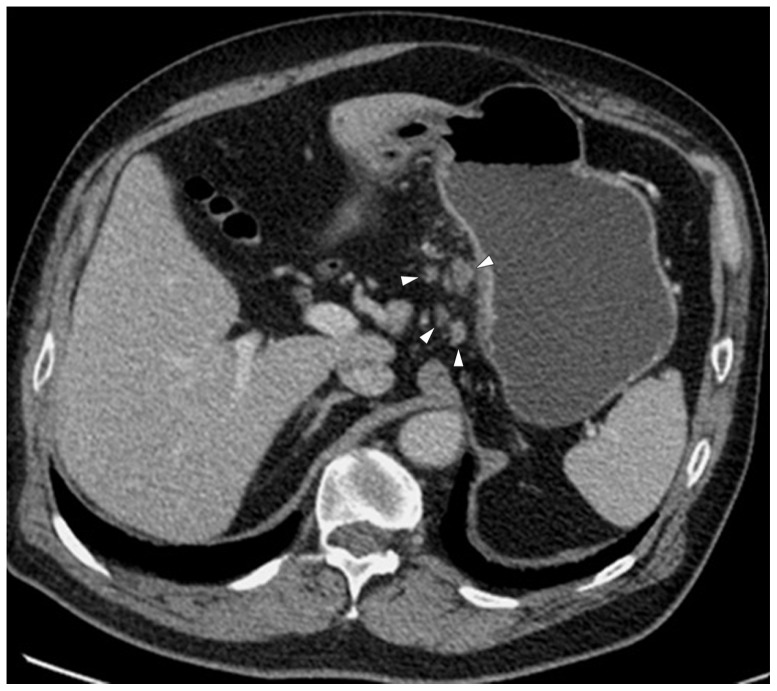
Same patient as Figure 3. Four lymph nodes (arrow heads) were detected with kVp image.

The comparisons for N staging are shown in Tables 4 and 5. The overall accuracies for N staging were 80.0% and 75.0% from the optimal keV images and the kVp images, respectively. The improvement of the overall accuracy in N staging using the keV images was statistically significant (p<0.05). The accuracy of CT for tumor staging with kVp images was 82.3% (88 of 96 patients) for N0, 79.4% (77 of 96 patients) for N1, 90.6% (87 of 96 patients) for N2, and 97.9% (94 of 96 patients) for N3 tumors. These values with the optimal keV monochromatic images were 85.4% (82 of 96 patients) for N0 tumors, 84.4% (81 of 96 patients) for N1, 91.7% (88 of 96 patients) for N2, and 98.9% (95 of 96 patients) for N3 tumors (Table 4). There was significant difference between them with McNemar test (p = 0.02) (Fig. 3, 4, 5, 6, 7, and 8). For patients with non metastatic disease, the accuracy, sensitivity and specificity for this distinction of N0 vs. N+ were 79.2%(65 of 82 patients), 88.8%(48 of 54 patients), 60.7%(17 of 28 patients) with kVp images and 82.9%(68 of 82 patients), 90.7%(49 of 54 patients), 67.9%(19 of 28 patients) with keV images (Table 5).

**Table 4:** Accuracies, sensitivities and specificities for N staging using kVp images (Group A) and optimal monochromatic images (Group B) with histological examination as the reference standard.

		Histological staging						
CT Staging		N0 (n = 28)	N1 (n = 43)	N2 (n = 23)	N3 (n = 2)	Accuracy (%)	Sensitivity (%)	Specificity (%)
A	N0	17	6	0	0	82.3	60.7	91.2
	N1	8	32	1	0	79.2	74.4	83.1
	N2	3	4	21	0	90.6	91.3	90.4
	N3	0	1	1	2	97.9	100	97.9
B	N0	19	5	0	0	85.4	67.9	92.6
	N1	6	35	1	0	84.4	81.4	86.8
	N2	3	3	21	0	91.7	91.3	91.8
	N3	0	0	1	2	98.9	100	98.9

N0–N3 data are presented as the number of patients; other variables as percentages.

**Table 5:** Accuracies, sensitivities and specificities for the distinction of N0 vs. N+ using kVp images (Group A) and optimal monochromatic images (Group B) with histological examination as the reference standard among patients with non metastatic disease.

		Histological staging				
CT Staging		N0 (n = 28)	N+ (n = 54)	Accuracy (%)	Sensitivity (%)	Specificity (%)
A	N0	17	6	79.2	88.8	60.7
	N+	11	48			
B	N0	19	5	82.9	90.7	67.9
	N+	9	49			

N0–N+ data are presented as the number of patients; other variables as percentages.

Fourteen cases were diagnosed as M1, including 6 cases of liver metastasis, 4 cases of peritoneal metastasis, 3 cases of distant lymph nodes involved and 1 case of pelvic metastasis. Among these cases, one case with peritoneal metastasis was missed by either the keV or kVp images. For M staging, both the keV and kVp images obtained the same accuracy, sensitivity, and specificity of 98.9% (95 of 96 patients), 92.85% (13 of 14 patients) and 100% (82 of 82 patients), respectively. (Table 6).

**Table 6:** Accuracies, sensitivities and specificities for M staging using kVp images (Group A) and optimal monochromatic images (Group B) with histological examination as the reference standard.

		Histological staging				
CT Staging		M0 (n = 82)	M1(n = 14)	Accuracy (%)	Sensitivity (%)	Specificity (%)
A	M0	82	1	98.9	92.8	100
	M1	0	13			
B	M0	82	1	98.9	92.8	100
	M1	0	13			

M0–M1 data are presented as the number of patients; other variables as percentages.

### Quantitative analysis of nIC

**Figure 9:**
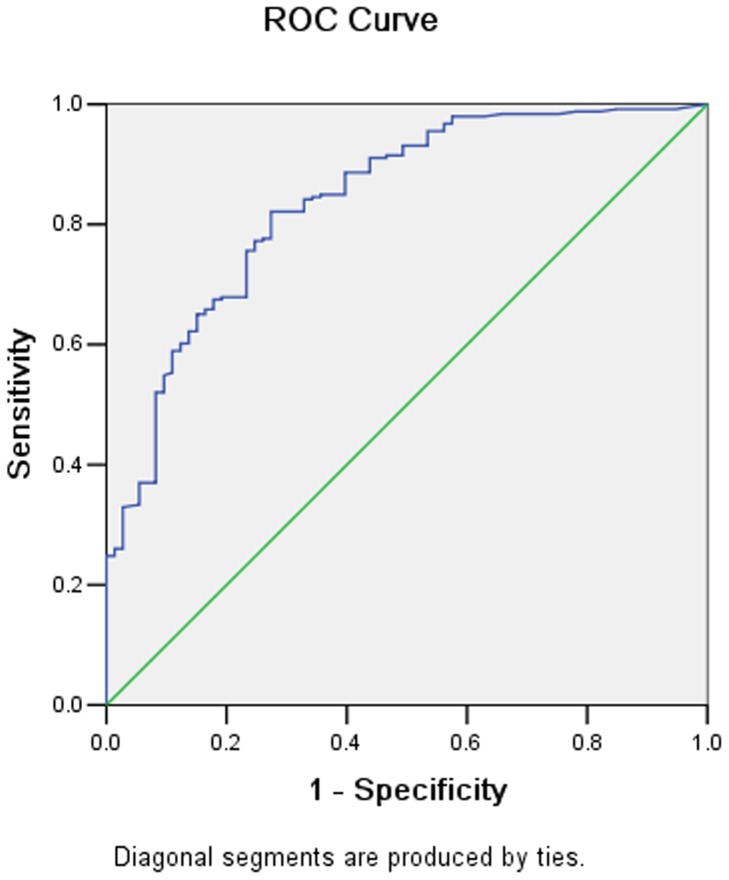
Receiver operating characteristic curves for differentiating metastatic and non-metastatic lymph node in arterial phase.

**Figure 10:**
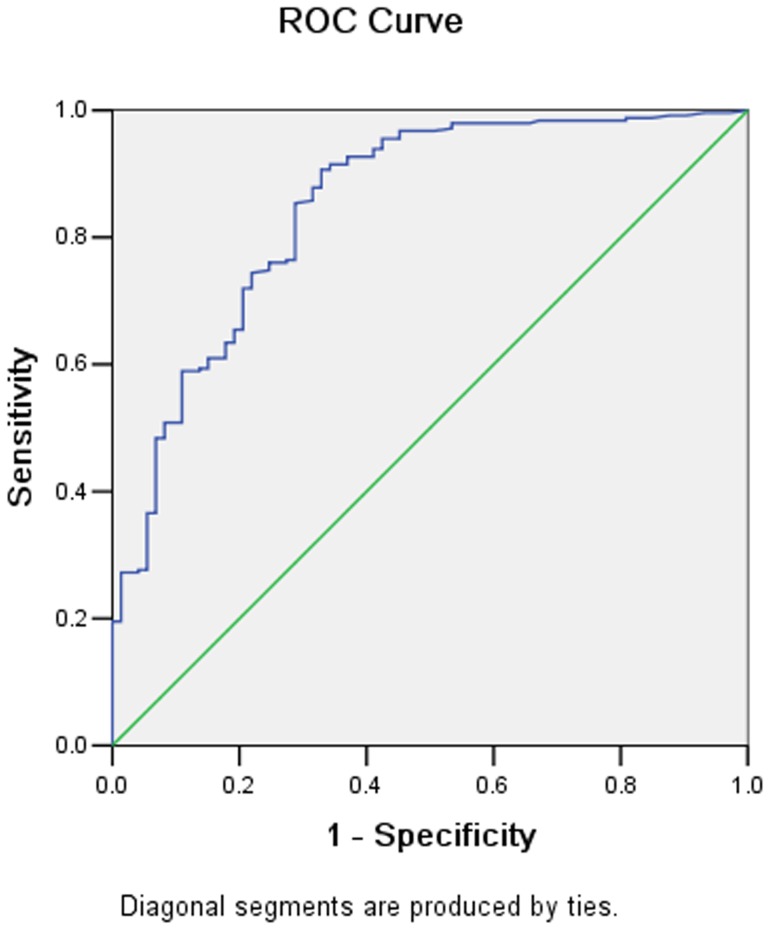
Receiver operating characteristic curves for differentiating metastatic and non-metastatic lymph node in portal phase.

**Figure 11:**
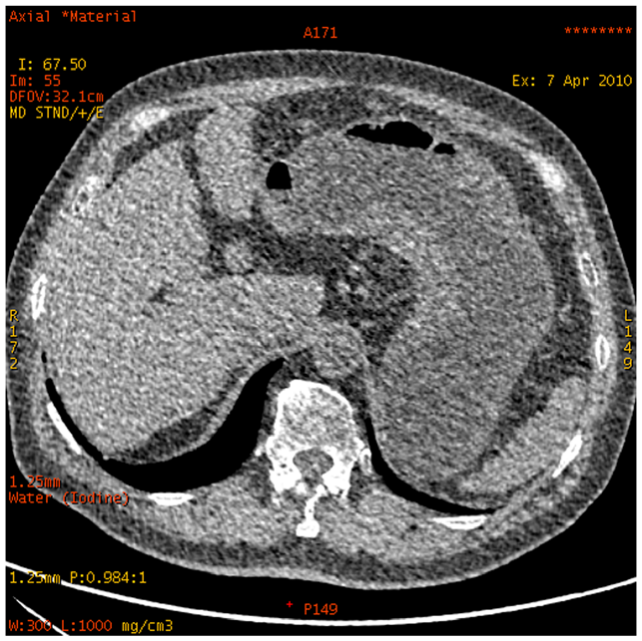
Water-based material decomposition images for a 62-year-old man with signet ring cell carcinoma obtained with dual energy spectral scan mode.

**Figure 12:**
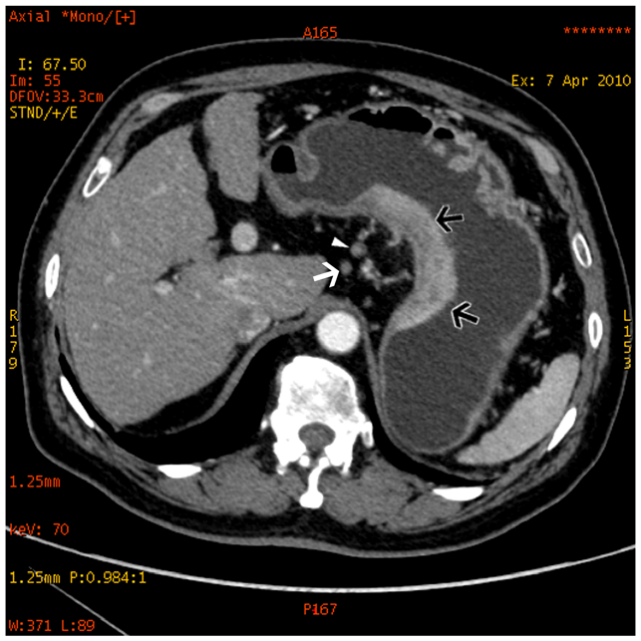
Same patient as Figure 11. Monochromatic image obtained at 70 keV energy level revealed the primary lesion (black arrows) and non-metastatic lymph node (arrow head) and metastatic lymph node (white arrow).

**Figure 13:**
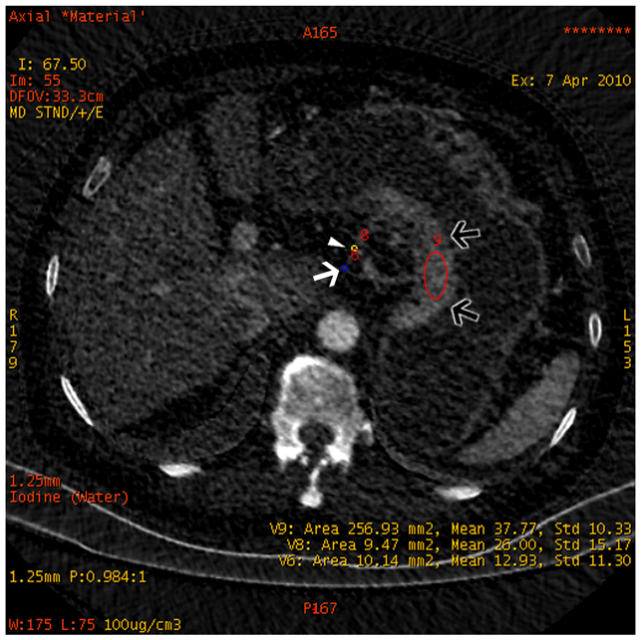
Iodine-based material decomposition images of same patient as Figure 11. The iodine concentration of the primary lesion (black arrows) was 37.77(mg/mL), non-metastatic lymph node (arrow head) was 26.00 mg/mL, and metastatic lymph node (white arrow) was 12.93 mg/ml.

**Figure 14:**
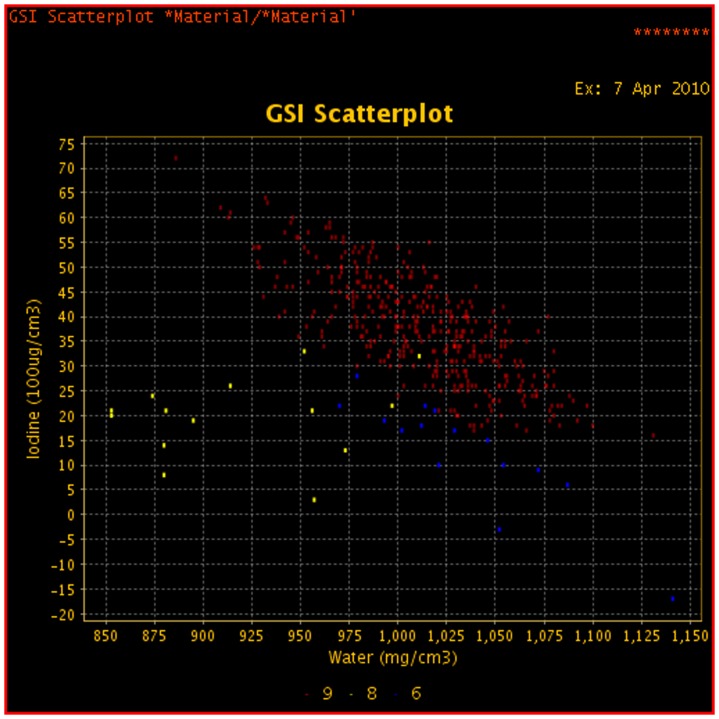
GSI scatterplot images water and iodine concentration plots for the same patient as Figure 11.

There were 68 adenocarcinoma, 16 signet ring cell carcinoma and 12 mucinous adenocarcinoma confirmed pathologically. The nIC values for these three types of cancers were 0.22±0.13, 0.23±0.13, and 0.24±0.09 in the arterial phase (AP), and 0.50±0.15, 0.52±0.23, and 0.48±0.15 in the portal venous phase (PP), respectively. There was no significant difference between them either in AP or in PP. However the nIC values for differentiated carcinoma and undifferentiated carcinoma was significantly different both in AP (mean 0.21±0.08 vs. 0.28±0.16, p = 0.02) and PP (0.54±0.17 vs. 0.46±0.12, p = 0.01). There were 246 lymph nodes proved to be metastatic and 73 lymph nodes to be normal. By using the receiver operating characteristic curves, the threshold values of nIC required to optimize both the sensitivity and the specificity for differentiating between the metastatic and non-metastatic lymph nodes were achieved (Fig. 9–10). The nIC values for the metastatic and non-metastatic lymph nodes were significantly different in AP (0.22±0.09 vs. 0.13±0.06, p<0.001) and PP (0.47±0.14 vs. 0.30±0.12, p<0.001) (Fig. 11, 12, 13, and 14). During AP, a threshold value of 0.145 for nIC would yield a sensitivity and specificity of 84.1% and 67.1% respectively. During PP, a threshold value of 0.333 for nIC would yield a sensitivity and specificity of 89.9% and 67.6% respectively.

Among the metastatic lymph nodes, the nIC values of signet-ring cell carcinoma were significantly different from adenocarcinoma (0.31±0.12 vs. 0.51±0.09, p = 0.02) and from mucinous adenocarcinoma (0.31±0.12 vs. 0.54±0.13, p = 0.01) in PP. No differences were found between the nIC for metastatic lymph nodes in adenocarcinoma and signet-ring cell carcinoma (0.24±0.09 vs. 0.15±0.07, p = 0.055) and in adenocarcinoma and mucinous adenocarcinoma (0.24±0.09 vs. 0.19±0.06, p = 0.32) (Table 7).

**Table 7:** Comparison of nIC in AP and PP with clinic-pathological characteristics of the patients including histological classification and metastic or nonmetastatic lymph nodes.

	Cases	nIC in AP	P value	nIC in PP	P value
Adenocarcinoma	68	0.23±0.11	0.96	0.50±0.15	0.81
Signet-ring cell carcinoma	16	0.23±0.13		0.52±0.23	
Mucinous adenocarcinoma	12	0.24±0.09		0.48±0.15	
Differentiated type	61	0.21±0.08	0.02	0.54±0.17	**<0.05**
Undifferentiated type	35	0.28±0.16		0.46±0.12	
Metastatic LN	246	0.22±0.09	**<0.001**	0.47±0.14	**<0.001**
Normal LN	73	0.13±0.06		0.30±0.12	
Metastatic LN					
-Adenocarcinoma	146	0.24±0.09	0.054 0.316	0.51±0.16	**0.021 0.01**
-Signet-ring cell carcinoma	52	0.15±0.07		0.31±0.12	
-Mucinous adenocarcinoma	48	0.19±0.06		0.54±0.13	

## Discussion

DEsCT imaging obtained with the single tube, rapid dual tube voltage switching technique provides the monochromatic images depicting how the imaged object would look if the X-ray source produced only single energy X-ray photons. This would allow for increased contrast resolution. It has been proved by some reports. According to Matsumoto's study, the DEsCT imaging at approximately 70 keV yielded lower image noise and higher CNR than the 120-kVp CT did for a given radiation dose [40]. Zhao indicated that both the image quality and CNR were improved for intra-hepatic and extra-hepatic portal veins with spectral CT imaging at 51 keV [41]. In addition, the use of a monochromatic x-ray beam in CT would reduce the beam-hardening artifacts and averaging attenuation effects commonly seen in the conventional CT scans with polychromatic x-ray beam [42]. The beam-hardening artifacts and averaging attenuation effects would sometimes cause attenuation values to be unreliable for verification of enhancing versus nonenhancing for small lesions [35], such as lymph nodes. In our study, the best keV value was found to be around 70 keV to provide the best contrast-noise-ratio for gastric cancers. The better image contrast resolution at the optimal energy level provided more accurate detection and measurement for the lymph node diameter and better differentiation between lymph nodes and small perigastric vessels.

In our study, the overall T staging was also improved from 73.9% with kVp images to 81.2% with the optimal monochromatic images, even though there was no significant difference between them (p = 0.153). The fact that the DEsCT scan mode allows the radiologists to choose an optimal imaging plane to accurately evaluate the depth of tumor invasion of the gastric wall and to identify the thin fat plane between the tumor and adjacent organs to avoid the partial-volume averaging effect. It improves the diagnostic accuracy for T staging, especially in T3-T4 (accuracy 85.4% and 91.7% respectively with optimal monochromatic images). In the present study, the T1 accuracy was 95.8%, which was slightly higher than previously reported. This may also be due in part to the low proportion of early tumors (9%) in this study.

It has been shown that the size of the lymph node is not a sufficient criterion to determine malignancy. Park considered a lymph node positive if the longest diameter was >1.0 cm or if it was 0.7 to 1.0 cm and showed strong enhancement, round shape, central necrosis, or perinodal infiltration, all of which suggest metastasis. Their results demonstrated an accuracy of 67.9% in T staging, and 56.9% in N staging [43]. GSI mode was adapted in our study and the diagnostic criteria by D'Elia F were followed [12]. Our study showed that the accuracy of CT in N staging was 75% with the kVp images and 80.0% with the optimal monochromatic images, revealing a significant difference (p = 0.015). The accuracies of CT for tumor staging with the kVp and optimal monochromatic images were 82.3%, 85.4% for N0 tumors, 79.2%, 84.4% for N1, 90.6%, 91.7% for N2, and 97.9%, 98.9% for N3, respectively. The reason for the promotion of accuracy with the monochromatic images may be due to the fact that the monochromatic images demonstrated better contrast resolution.

Even though the clearer images could be helpful to improve the accuracy of N staging, CT is relatively insensitive and also nonspecific for detecting nodal metastases due to its inability to detect microscopic nodal invasion, which is common in gastric cancer, and the presence of reactive nodes that may be greater than 10 mm [2]. How to quantitatively define the pathology of lymph nodes and how to predict the prognosis remain to be the challenges. On the basis of our results, DEsCT may offer us an encouraging alternative to indicate the vascular physiology in gastric tumors. Knowing how a substance behaves at two different energies can provide information about tissue composition beyond that obtainable with single-energy techniques [44]. The DEsCT is capable of extracting quantitative information about the elemental and molecular composition of tissue and contrast materials basing on their attenuation properties. Water and iodine are often selected as the basis pair for material-decomposition image presentation because their atomic numbers span the range of atomic numbers for materials generally found in medical imaging and approximate those of soft tissue and iodinated contrast material to result in material-attenuation images that are intuitive to interpret. The iodine concentration in lesions derived from the iodine-based material decomposition images is quantitative, and thus might be a useful parameter [35], [42].

In our study, DEsCT was performed and normalized iodine concentration was detected both in the lesions and in the lymph nodes. Basing on our data, there were 246 lymph nodes proved to be metastatic and 73 lymph nodes to be non-metastatic. The nIC values for metastatic and non-metastatic lymph nodes were significantly different in AP (0.22±0.09 vs. 0.13±0.06, p<0.001) and PP (0.47±0.14 vs. 0.30±0.12, p<0.001). Among the metastatic lymph nodes, the nIC values of signet-ring cell carcinoma were significantly different from adenocarcinoma (mean 0.31±0.12 vs. 0.51±0.09, p = 0.02) and from mucinous adenocarcinoma (mean 0.31±0.12 vs. 0.54±0.13, p = 0.01) in PP. The characteristics of signet ring cell carcinoma are its potential to diffusely infiltrate the gastric wall, to cause a marked scirrhous reaction and the prognosis of patients with advanced signet ring cell carcinoma was poor compared with patients with other types of gastric cancer [45]. nIC of the metastatic lymph nodes from signet ring cell carcinoma appears to be lower compared to other types while the nIC of the primary lesions of signet ring cell carcinoma were higher than others. We speculate that the reason is due to the fact that most of the lesion of signet ring cell adenocarcinoma shows diffusely infiltrative growth of malignant cell groups intermingled with mature and immature fibrosis, containing abundant fibroblasts and neovascularity [46]. Compared with the primary lesion, metastatic lymph nodes could be with fewer immature fibrosis.

Another finding is that the nIC values for differentiated carcinoma and undifferentiated carcinoma appeared to be significantly different both in AP (mean 0.21±0.08 vs. 0.28±0.16, p = 0.02) and PP (0.54±0.17 vs. 0.46±0.12, p = 0.014). Although minimal infiltration of cancer cells into the deeper layer, observed often in poorly differentiated type tumors, is beyond the resolution of MDCT [47], it is likely that quantitative measurement could be helpful. During the portal phase (70 s after the contrast injection), the contrast media is supposed be diffused into the lesion just like the end stage of perfusion, during which the enhancement of the tumor is caused by the contrast media both in intrasvascular and extravascular space with the leakage to extravascular space [33]. It might be the best time point to do the analysis. Having demonstrated the ability to distinguish between benign and malignant lymph nodes, and between different pathological classifications, nIC attracts our attention. However whether it could be a novel independent prognostic indicator remains an uncertainty and further investigation should be implemented.

Radiation dose was also something worth mentioning. In the standard clinical protocol with conventional multislice CT, three-phasic enhancement scans were often performed: the arterial phase was used for lesion detection, the portal venous phase was used to differentiate the stomach from adjacent organs and lymph node evaluations, and the delayed phase was used to help evaluate the depth of gastric wall invasion [48]–[49]. As metastatic hepatic lesions are usually hypovascular, the optimal CT strategy is helical scanning during the portal venous phase of enhancement. This technique improves lesion identification by increasing the attenuation of normal liver tissue, and occasionally rim enhancement of a hypoattenuating metastasis can be seen [50]–[51]. On the basis of our data, dual-phasic, DEsCT imaging could be an alternative to the conventional three-phasic scans as the accuracy of TNM staging with the dual-phasic spectral imaging was equivalent to or a little bit superior to the previous studies. In addition, nIC of both the primary lesions with different histology and metastatic or non-metastatic lymph nodes appeared to be statistically different, providing additional quantitative information for differential diagnosis. Our total radiation dose was 43.68 mGy for the two enhanced dual energy spectral scans which was comparable to the total dose of about 50 mGy administered for a conventional three-phase abdominal CTA in a normal-size patient at our institution on a 64-slice CT scanner.

There are several limitations in our study. First, since the spectral CT images provided additional information and required special image viewer, it was impossible to be completely blinded to the imaging methods if the same radiologist was reviewing both the conventional kVp and monochromatic keV images. Therefore four radiologists were divided into two groups for the conventional and monochromatic keV images separately. These four radiologists were all specialized in gastrointestinal imaging, and they have been working on TNM staging of gastric cancer in MDCT for over ten years. They all took part in the paper published on TNM staging [52]. However, even though care was taken in terms of mixing the radiologists with clinical experiences and giving the same instruction for the cancer staging, differences of judgment between various people could still exist and this may have impact on the final results. Second, to reduce the uncertainty for comparing the lymph nodes one-to-one between CT images and surgery, the metastatic lymph nodes with size less than 6 mm on CT were not included in our study. Though an analysis of 1082 nodes resected in patients with gastric cancer who were examined preoperatively with spiral CT (5-mm section thickness) revealed that only 21% of nodes 5–9 mm were positive [19], another study of nodes resected at surgery [53] indicated that 55% of metastatic lymph nodes were less than 5 mm in diameter. The exclusion of lymph nodes less than 5 mm may bias the cutoff values for nIC for the population of metastatic lymph nodes. Third, in this study, the DEsCT scans were performed on the first generation Discovery CT750HD scanner in 2010 with a fixed mA value of 600 mA, which yielded a CT dose index volume (CTDIvol) of 21.84 mGy. Even though this value was comparable to the dose (single enhanced scan at about 20 mGy) administered for a conventional abdominal CTA in a normal-size patient at our institution on a 64-slice CT scanner as mentioned in the previous paragraph, it is considered a bit high in today's environment. With the introduction of the second generation of CT750HD, we have seen dose reduction of 30% for the GSI mode, and further dose reduction is forthcoming. Forth, since DESCT is a brand new technology in the TNM staging of gastric cancer and our research is being devoted to evaluate its ability. It is premature to change the management using this preliminary results.

In conclusion, DEsCT imaging provides both the monochromatic images and material-decomposition images. The monochromatic images may be used to improve the N staging accuracy for gastric cancers, and the quantitative iodine concentration measurements from the material- decomposition images may be helpful for differentiating between differentiated type and undifferentiated type of carcinoma, and between metastatic and non-metastatic lymph nodes. DEsCT may provide new opportunities for detailed preoperative evaluation of not only the gastric morphology but also the vascularity for primary lesions and lymph nodes.

## Supporting Information

Appendix S1 **The gender, age, pathological staging and treatment of the 96 patients were reported in the Appendix S1.**
(DOC)
