# Supplementary material for: Gastric Cancer Staging with Dual Energy Spectral CT Imaging
Source: PLoS One. 2013 Feb 12;8(2):e53651. doi: 10.1371/journal.pone.0053651 (PMC3570537; doi:10.1371/journal.pone.0053651)
Supplement: Appendix S1 — The gender, age, pathological staging and treatment of the 96 patients were reported in the Appendix S1. (DOC) [file pone.0053651.s001.doc]

S Appendix

| **Gender** | **Age** | **Pathological staging** | **Treatment** |
| --- | --- | --- | --- |
| m | 71 | T3N1M0 | D2 |
| m | 48 | T2N0M0 | D2 |
| m | 61 | T3N1M0 | D2 |
| f | 62 | T3N1M0 | D2 |
| m | 36 | T4N2M0 | palliative resection |
| m | 60 | T3N1M0 | D2 |
| f | 28 | TisN0M0 | D1 |
| m | 58 | T2N0M0 | D2 |
| f | 47 | T3N0M0 | D2 |
| f | 68 | T2N1M0 | D2 |
| m | 69 | T2N1M0 | D2 |
| m | 71 | T3N1M0 | D2 |
| m | 68 | T3N1M0 | D2 |
| m | 48 | T3N1M0 | D2 |
| m | 66 | TisN0M0 | D1 |
| m | 61 | T3N0M0 | D2 |
| m | 39 | T3N0M0 | D2 |
| f | 54 | T4N1M1 | palliative resection |
| m | 47 | T3N2M0 | D2 |
| f | 31 | T2N0M0 | D2 |
| m | 74 | T3N2M0 | D2 |
| f | 62 | T3N1M0 | D2 |
| m | 48 | TisN0M0 | D2 |
| m | 42 | T3N1M0 | D2 |
| f | 55 | T4N3M1 | palliative resection |
| f | 36 | T3N0M0 | D2 |
| m | 60 | T3N1M0 | D2 |
| f | 42 | T3N0M0 | D2 |
| m | 78 | T4N2M1 | palliative resection |
| m | 66 | T2N0M0 | D2 |
| m | 73 | T1N0M0 | D2 |
| m | 59 | T1N0M0 | D2 |
| m | 63 | T3N1M0 | D2 |
| f | 37 | T2N0M0 | D2 |
| f | 54 | T3N1M0 | D2 |
| m | 53 | T3N1M0 | D2 |
| m | 48 | T4N1MO | D2 |
| m | 59 | T4N2M1 | palliative resection |
| f | 69 | T2N2M0 | D2 |
| f | 40 | T1N0M0 | D2 |
| f | 47 | T2N0M0 | D2 |
| f | 52 | T3N2M0 | D2 |
| m | 70 | T3N2M0 | D2 |
| m | 68 | T3N1M0 | D2 |
| m | 60 | T3N1M0 | D2 |
| f | 56 | T3N1M0 | D2 |
| f | 46 | T3N2M0 | D2 |
| m | 59 | T3N1M0 | D2 |
| m | 77 | T3N1M0 | D2 |
| f | 47 | T2N0M0 | D2 |
| m | 47 | T3N1M0 | D2 |
| m | 38 | T2N0M0 | D2 |
| f | 61 | T3N1M0 | D2 |
| m | 58 | T3N2M0 | D2 |
| m | 69 | T3N1M0 | D2 |
| m | 63 | T2N1M0 | D2 |
| m | 72 | T3N2M0 | D2 |
| m | 57 | T3N1M0 | D2 |
| m | 55 | T3N2M1 | palliative resection |
| m | 72 | T3N2M0 | D2 |
| f | 75 | T4N1M0 | D2 |
| m | 37 | T3N2M0 | D2 |
| m | 72 | T2N0M0 | D2 |
| f | 49 | T4N1M1 | palliative resection |
| m | 76 | T4N2M1 | palliative resection |
| m | 76 | T4N2M0 | D2 |
| m | 68 | T4N1M0 | D2 |
| f | 68 | T3N1M0 | D2 |
| f | 47 | T2N0M0 | D2 |
| m | 57 | T3N3M1 | palliative resection |
| f | 54 | T1N0MO | D2 |
| m | 35 | T2N1M0 | D2 |
| m | 39 | T2N0M0 | D2 |
| f | 47 | T1N0M0 | D2 |
| m | 62 | T1N0M0 | D2 |
| f | 45 | T4N2M1 | palliative resection |
| f | 35 | T2N0M0 | D2 |
| f | 41 | T3N1M0 | D2 |
| m | 66 | T4N1M1 | palliative resection |
| f | 47 | T3N1M0 | D2 |
| m | 60 | T2N0M0 | D2 |
| f | 78 | T4N1M0 | D2 |
| m | 71 | T3N2M1 | palliative resection |
| m | 77 | T4N2M0 | D2 |
| f | 60 | T4N2M1 | palliative resection |
| f | 51 | T3N1M0 | D2 |
| m | 52 | T2N1M0 | D2 |
| f | 59 | T4N2M1 | palliative resection |
| m | 63 | T4N1M0 | D2 |
| m | 62 | T3N1M0 | D2 |
| f | 53 | T3N0M0 | D2 |
| m | 30 | T3N2M0 | D2 |
| m | 62 | T4N2M1 | palliative resection |
| m | 66 | T4N1M0 | D2 |
| f | 43 | T3N1M0 | D2 |
| f | 53 | T3N1M0 | D2 |
